# Development of a combinatory filtration system for pollution and virus abatement by optimized nanoparticle deposition

**DOI:** 10.1371/journal.pone.0264991

**Published:** 2022-03-31

**Authors:** Ishika Nag

**Affiliations:** Seminole State College, Sanford, FL, United States of America; Tsinghua University, CHINA

## Abstract

PM_2.5_, particulate matter less than 2.5 microns, is the leading contributor to air pollution which results in cardio-vascular and respiratory diseases. Recent studies also indicate a strong correlation between ambient air pollution and COVID-19 cases, which have affected the lives of billions of people globally. Abatement technologies such as ionic and other high efficiency filtration systems are expensive and unaffordable in communities with limited resources. The goal of this study was to develop a mask with an optimized nanoparticle coating which has a dual capability of particulate matter and virus filtration, while being affordable and safe for human use. The nanoparticles were selected for their filtration and virucidal capabilities. Particle filtration efficiency, tested with a wind tunnel and PM_2.5_ from incense sticks measured by laser particle detectors, improved by ~60% with nanoparticle coatings on KN95 and surgical masks. Virus filtration efficiency, tested using nebulized NaCl particles as a virus surrogate, improved by 95% with coated masks. The nanoparticle retention efficacy, tested by simulating a normal 8-hour workday, was well within the permissible exposure limits. This technology has several applications such as in personal protective equipment for virus protection, and in air-conditioning and car cabin filters for pollution abatement. In conclusion, the chosen combination of nanoparticles provides an effective and safe solution for both particulate matter and viral particle filtration.

## 1. Introduction

Particulate matter (PM) is one of the main causes of air pollution, consisting of a mixture of solid and liquid particles suspended in the air. The most used health indicator related to PM refers to the mass concentration of particles with a diameter less than 2.5μm, PM_2.5_ [1,2]. Primary sources of PM_2.5_ include automobile emissions, household fuel [3] and waste burning, energy production from fossil fuels [4], and industrial activities such as construction, mining, cement production, etc. The most polluted areas around the world tend to be in developing countries in South-East Asia, Africa, and China, due to their increased density of urban population, significant use of fossil fuels and relatively inadequate control measures [5].

The majority (91%) of the world’s population lives in places exceeding the World Health Organization’s air quality guidelines and 7 million people die every year because of air pollution [5]. The primary causes of such premature deaths are pulmonary and heart disease, stroke, lung cancer, and acute respiratory infections in children. PM_2.5_, due to its small size, is capable of penetrating deep into lung passageways and entering the bloodstream causing cardiovascular, cerebrovascular, and respiratory impacts [6,7]. Furthermore, long term exposure to air pollution has been found to increase the vulnerability to the most severe impacts of coronavirus outbreaks such as SARS in 2003 and SARS-CoV-2 in 2019 [8–11]. Similarly, an increase of only 1 μg/m^3^ in PM_2.5_ is associated with an 11% increase in the COVID-19 death rate [9].

Abatement technologies, such as ionic [12] and high efficiency particulate air (HEPA) filtration systems [12], have been developed that can filter PM_2.5_ particles significantly but remain to be quite expensive, and unaffordable to communities with limited resources [13]. Therefore, a cost-effective and efficient abatement system is essential to help resolve this issue.

Nanoparticles have a high surface to volume ratio, which enhances entrapment of particulate matter by their diffusion and electrostatic attraction mechanisms. Nanofiber membranes have been developed for both indoor and outdoor air protection by electrospun synthesized polyacrylonitrile:TiO_2_ [14]. Graphene oxide aerogels with a special porous structure have been developed which combine the advantages of its high adsorption alongside its mechanical properties, and have demonstrated excellent performance in the capture of PM_2.5_ particles [15]. An integrated fiber mop and floor lamp with TiO_2_ nanoparticles have been developed to utilize the photocatalytic properties for increasing air purification performance [16]. TiO_2_ particles have been synthesized by a sol–gel procedure and deposited on a porous quartz tube to manufacture a photocatalytic filter tube [17]. However, it has also been highlighted that the available research has some shortcomings in personal protective equipment related to comfort, safety, and functional integration [18]. Hence, a simple application technique of nanoparticles, selected based on their filtration, virucidal and non-toxicity, onto various filtration systems can provide an affordable and comparable alternative to expensive high quality air filtration devices.

The nanoparticles used for this experiment like graphene, titanium dioxide (TiO_2_), and zinc oxide (ZnO), are known to have filtration properties due to their high adsorption capabilities [19–22]. TiO_2_ and ZnO nanoparticles (NP), with their photocatalytic properties, absorb the ultraviolet component of sunlight which excites the electrons (e-) from Valence Band (VB) to Conduction Band (CB) and act as a catalyst to form the superoxide anion (O2•-) and reactive hydroxyl (OH•) radicals from atmospheric moisture and oxygen (1). These radicals react with the PM_2.5_ particles due to their strong oxidizing capabilities converting them into CO_2_ and H_2_O [18].


$$\begin{array}{l} 1)\;\mathrm{NP}(e‐\mathrm{CB})+O_{2}\to \left(O_{2}•‐\right)+\mathrm{NP}[The\;photo\;generated\;(e‐)\;reacts\;with\;adsorbed\;O_{2}\;to\;form\;superoxide\;radical(O2•‐)]\\ 2)\;\left(O_{2}•‐\right)+H_{2}O\to {\mathrm{HO}}_{2}•+\mathrm{OH}‐\\ 3)\;{\mathrm{HO}}_{2}•+H_{2}O\to \mathrm{OH}•+H_{2}O_{2}\\ 4)\;H_{2}O_{2}\to 2\mathrm{OH}•\;[The(O_{2}•‐)in\;turn\;reacts\;with\;moisture(H_{2}O)\;to\;form\;(OH•)\;hydroxyl\;radical]\\ 5)\;\mathrm{OH}•+\mathrm{air}\;\mathrm{pollutant}\to {\mathrm{CO}}_{2}+H_{2}O[The\;(OH•)degrades\;pollutonts\;to\;CO_{2}\;and\;H_{2}O] \end{array}$$ (1)

Metal based nanoparticles, like CuO and ZnO, have unique physico-chemical properties which enable them to interact with viruses [17], and have been added to the admixture of nanoparticles in this study [19]. Respiratory diseases such as COPD, bronchitis, and asthma lead to the overexpression of the angiotensin-converting enzyme 2 (ACE2) receiver in human respiratory cells for viral attachment. The SARS-CoV-2 virus primarily attacks the respiratory tract. Its spike protein attaches to the overexpressed ACE2 receptors in the epithelial cells of the tract, thus causing respiratory disorders [23]. These metal-based nanoparticles generate Reactive Oxygen Species which oxidize viral proteins and nucleic acids, such as the spike protein in SARS-CoV-2 virus [17].

The objectives of the current work are to improve the Particulate Filtration Efficiency (PFE) and the Virus Filtration Efficiency (VFE) of a regular mask by the impregnation of an optimized mixture of nanomaterials, while demonstrating that the Nanoparticle Retention Efficacy (NRE) are within acceptable Permissible Exposure Limits (PEL), typically between 10–15 mg/m^3^, as defined by the Occupational Safety and Health Administration (OSHA) [24]. Furthermore, the goal of this work is to develop a simple application technique of the nanoparticles such that it can be applied to various filtration systems in different parts of the world, thus providing an affordable and comparable alternative to expensive high quality air filtration devices.

## 2. Materials and methods

### 2.1. Nanoparticle deposition method

The nanoparticles used for this study were Titanium Dioxide (TiO_2_ Anatase, 99.5% 40nm, US Research Nanomaterials, Inc.(USRNI)), Zinc Oxide (ZnO, 99+%, 35–45 nm, USRNI), Graphene (Alfa Aesar™ nanoplatelets aggregates, S.A. 500m^2^/g, Fisher-Scientific), Silicon Dioxide (SiO_2_, 99.5+%, 15-20nm, P-type, Porous, USRNI), and Copper Oxide (CuO, 99%, 40nm, USRNI).

**Fig 1:**
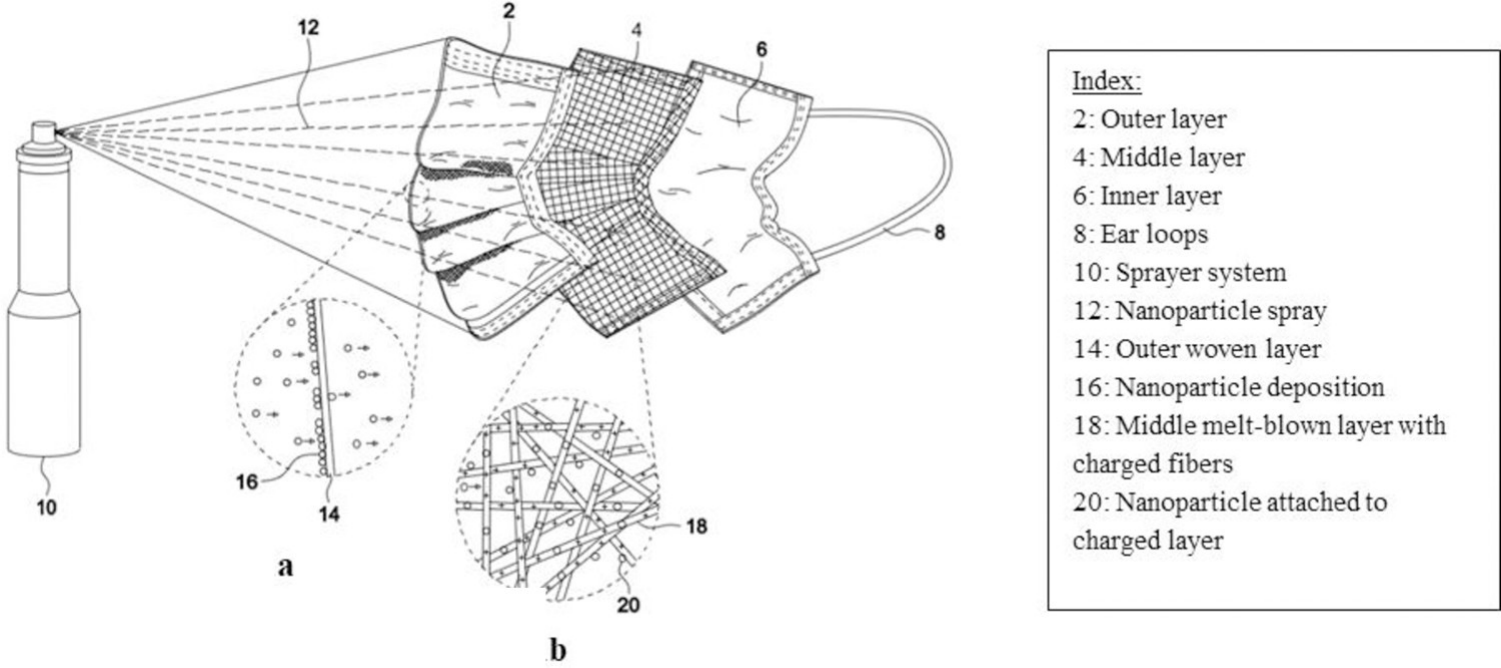
A typical three-layered surgical mask being embedded with nanoparticles with a pressurized sprayer coating technique.

The combination of nanoparticles was mixed with ethanol (200 Proof (100%), USP/EP/ACS, Fisher-Scientific) to create a suspension which appears as a slurry. This suspension was then aerosolized using the pressurized sprayer system (Preval Airless Paint Sprayer, 70 psi), and the aerosolized spray was directed towards the masks while maintaining a spray-distance of about 15–18 cm (Fig 4) The masks were air-dried for at least 8 hours and then tested for efficacy. Some nanoparticles were deposited on the outer non-woven layer (Fig 1A) while others penetrated onto the melt-blown middle layer with positively charged fibers due to the force of the pressurized sprayer system and attached onto the fibers of this middle layer (Fig 1B).

### 2.2. Surface morphology of nanoparticle coatings

**Fig 2:**
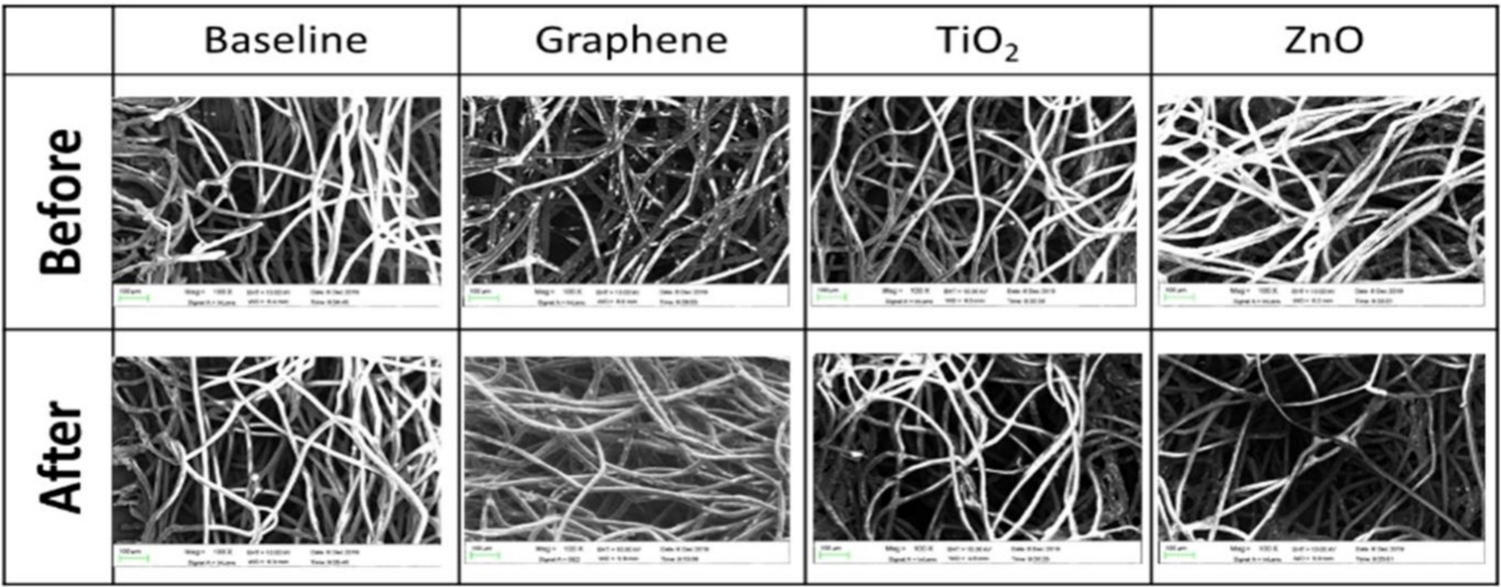
Scanning Electron Microscope (SEM) images of uncoated and coated masks, before and after exposure to PM_2.5_ particles. The ‘before’ images confirm the adhesion of the nanoparticles to the fibers of the filtration media. The ‘after’ images demonstrate the adsorption of the PM_2.5_ particles onto the nanoparticles and the fibers.

The surface morphology of the filters was characterized using the scanning electron microscopy (SEM) imaging technique. SEM images (Fig 2) enabled the confirmation of the nanoparticle adhesion to the fibers of the masks in the ‘before’ images and the entrapment of particulate matter onto the nanoparticle surfaces in the ‘after’ images of the coated masks. The embedded nanoparticles enhances the diffusion and electrostatic attraction mechanisms of filtration due to high surface to volume ratio and photocatalytic activation properties.

### 2.3. Experimental set-up

**Fig 3:**
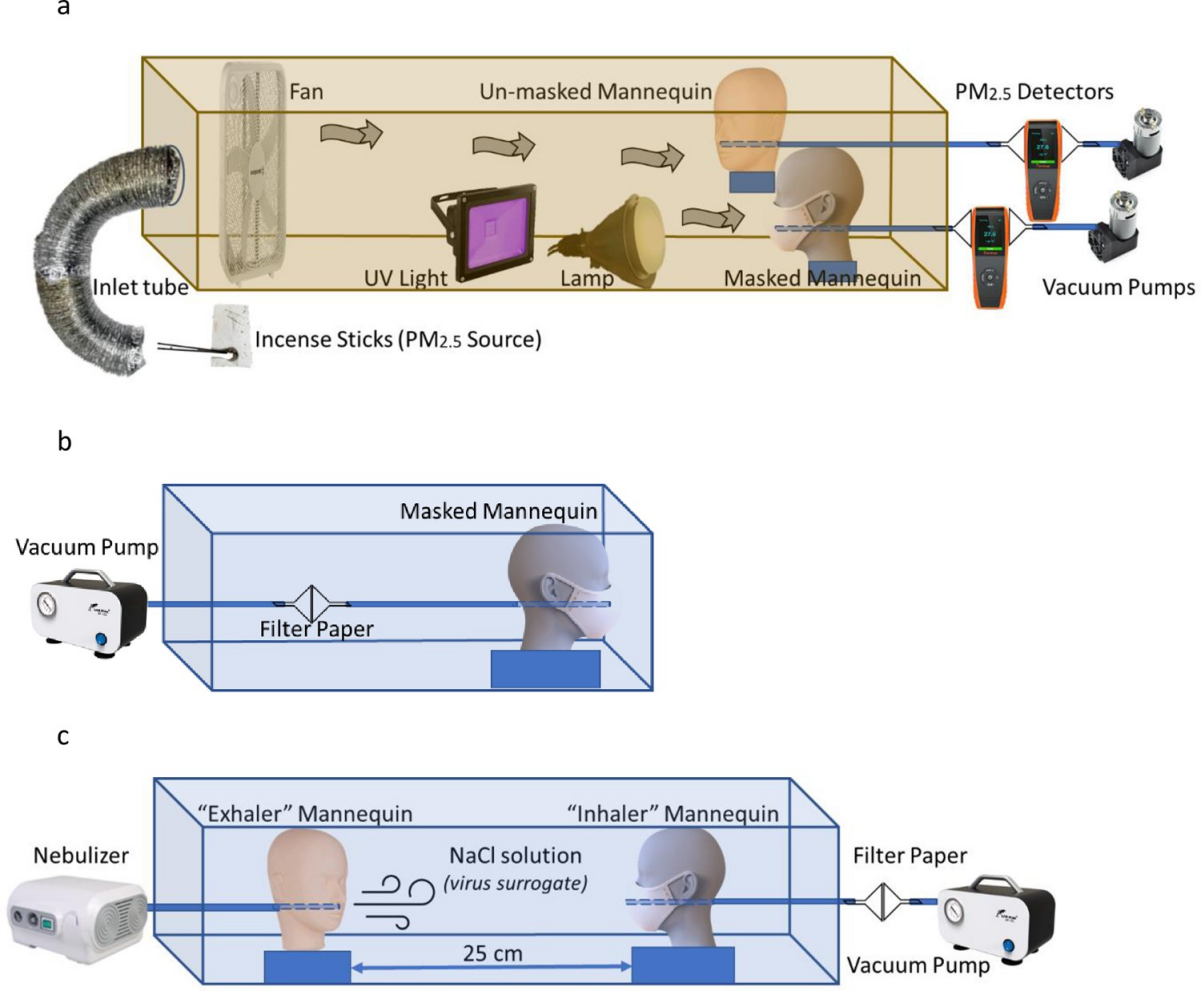
a. The PFE experimental set-up included a wind tunnel with a fan to create the draft, incense sticks to simulate the PM_2.5_, laser particle detectors to measure the filtration efficiency between masked and unmasked mannequins, with vacuum pumps providing the negative pressure for breathing simulation. b. The NRE experimental set-up, with a vacuum pump simulated 8 hours of continuous breathing and a fine (pore Ø 9 nm) filter paper collected dislodged nanoparticles from the coated masks. c. VFE set-up with an “inhaler” mannequin in front of another mannequin exhaled nebulized NaCl particles as a surrogate for virions encased in respiratory droplets.

A wind tunnel was designed to test for the Particulate Filtration Efficiency (PFE) of the masks (Fig 3A). A cardboard box was used as the body of the tunnel, with an aluminum exhaust tube connecting the PM source to the inlet section of the tunnel, and plexiglass windows for visualization of the detector readings. Incense sticks were used as the PM_2.5_ source [25,26] and a fan (Lasko, 50x50 cm) placed inside the tunnel, created the draft. Two soft silicone mannequin heads (Yephets, 23x15x10 cm), one with a nanoparticle coated mask and another control mannequin without a mask were tested side by side with vacuum pumps (HSH-Flow, 6W, 8L/min, 120 KPa) simulating human breathing. Laser particle detectors (Temtop, LKC-1000S) were connected by plastic tubing (Ø 0.64 cm) and funnels (Ø 7 cm) to the mannequins and to the vacuum pump, to measure PM_2.5_. A manometer (PerfectPrime, AR1890P2) was used to measure pressure drop, a 5000K lamp (Hyperikon, 15W) was used to simulate daylight and a UV lamp (Houlight, UV-A, 385–400 nm, 10W) was used to activate the photocatalytic properties of the nanoparticles. Two different types of masks–KN95 (ChiSip, 5-layer) and Surgical (Wapike, 3-layer disposable)–were tested with nanoparticles, and without any nanoparticles as controls. PFE was evaluated (2) by measuring the flow rate (mg/m^3^) of PM_2.5_, of the masked mannequin (φ_2_) and the control mannequin without mask (φ_1_); while both mannequins were placed side-by-side and exposed to the same environment.


$$\begin{array}{l} PFE(\%)=\frac{\varphi_{1‐}\varphi_{2}}{\varphi_{1}}\times 100\\ \varphi_{1}=PM_{2.5}\;without\;mask\\ \varphi_{2}=PM_{2.5}\;with\;mask \end{array}$$ (2)

Nanoparticle Retention Efficacy (NRE) was tested by connecting the mannequin wearing the nanoparticle coated mask to a vacuum pump simulating continuous human breathing (Fig 3B). The dislodged nanoparticles were collected on a fine filter paper (Supertek, Grade 1, Ø 110 mm, 9 nm pore Ø), encased in a collection chamber using two funnels and sealed with a gasket (Plumb Pak, Ø 3.8cm) and circumferentially attached clips (Acco, 3.2 cm). A sealing test was performed using the soap bubble leak test. The set-up was housed in a plexiglass enclosure (61x30.5x30.5 cm) to protect against wind draft and maintain consistency.

NRE was used to evaluate whether the dislodged nanoparticles from the mask, if inhaled, were still within the PEL as specified by OSHA [24]. As seen in (3), the PEL Utilization was calculated by comparing the weight of nanoparticles dislodged from the mask during an 8-hour operational period (ω_i_) to the PEL limit for that nanoparticle (ω_iPEL_) and taking a weighted average of the nanoparticles embedded in that mask. The total weight of all the nanoparticles collected was measured using the gravimetric method with a micro balance (0.1 mg accuracy), as recommended in the Center for Disease Control (CDC) test procedure [28] and the individual weights of the nanoparticles were calculated by the ratio of their molecular weights.


$$PEL\;Utilization\;(\%)=\frac{1}{n}\times \overset{n}{\underset{i=1}{\sum }}\frac{\omega_{i}÷3.84}{\omega_{i_{PEL}}}\times 100\begin{array}{lll} n & = & number\;of\;NPs\;in\;mask\\ i & = & NP\;type\;\left(e.g.TiO_{2},ZnO\;etc.\;\right)\\ \omega_{i} & = & weight\;of\;NP_{i}\;inhaled\;from\;mask\;[mg]\\ \omega_{i_{PEL}} & = & Permissible\;Exposure\;Limit\;for\;NP_{i}\left[mg/m^{3}\right]\\ 3.84 & = & Total\;volume\;of\;air\;breathed\;in\;8\;hours\;\left[m^{3}\right]\\ & = & 8\;[l/\min]\times 60\;[\min]\times 8\;[hours]\times 10^{-3}\left[m^{3}\right] \end{array}$$ (3)

Virus Filtration Efficiency (VFE) was tested (Fig 3C) with nebulized NaCl particles (Ø 0.5–10 μm) as a surrogate for virus charged respiratory droplets, per the test protocol recommended by (CDC) [27], which are typically 1–5 μm in diameter [29]. A mannequin connected to the nebulizer (Mayluck, 0.25 ml/min atomization rate, 0.5–10 μm particle size) ‘exhaled’ the NaCl particles (0.9% saline solution made from distilled water and table salt) which were ‘inhaled’ by the mannequin wearing the nanoparticle coated mask. The mannequins were kept 25 cm apart and the exposure time was 20 mins per mask, in order to get enough NaCl deposited in the collection chamber to be quantified by the gravimetric measurement procedure. The set-up was housed in a plexiglass enclosure (122x30.5x30.5 cm) to protect against wind draft and maintain consistency. The Virus Filtration Efficiency (VFE) was calculated (4) by comparing the weight of NaCl deposited on the fine filter paper after being inhaled through the mask (γ_2_), to the control case without mask (γ_1_).


$$\begin{array}{l} VFE(\%)=\frac{\gamma_{1‐}\gamma_{2}}{\gamma_{1}}\times 100\\ \gamma_{1}=NaCl\;(virus\;surrogate)\;inhaled\;without\;mask\;\\ \gamma_{2}=NaCl\;(virus\;surrogate)\;inhaled\;with\;mask\; \end{array}$$ (4)

### 2.4. Viral load calculation

**Fig 4:**
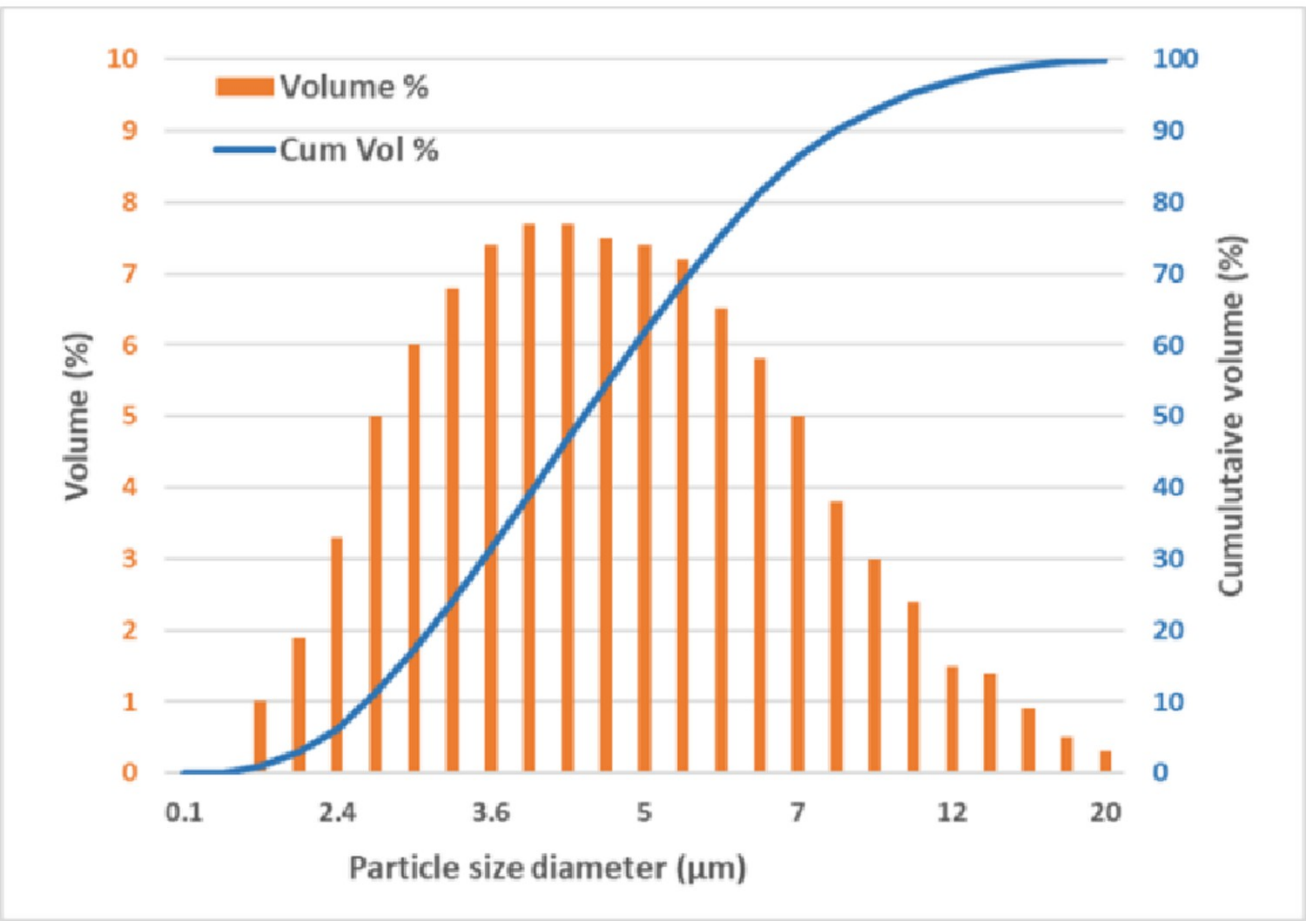
The distribution of nebulized NaCl particles with a median mass aerodynamic diameter (MMAD) of 5.3 μm is representative of typical aerosolized virus particle droplets.

Studies on respiratory droplet sizes [30] have indicated particle sizes between 0.8 to 5 μm, where breathing produced droplet sizes of 0.8 μm at an average concentration of 0.1 cm^-3^ while vocalization (speech) produced droplet sizes between 3.5–5 μm at an average concentration of 1.1 cm^-3^. The nebulizer used for this study produced aerosolized droplet sizes with a median mass aerodynamic diameter (MMAD) of 5.3 μm, as seen in Fig 4, which correlates well with typical sizes of aerosolized respiratory droplets, carrying different virions. The typical particle concentration, for breathing or vocalization, is characterized between 0.1–1.1 cm^-3^ at a typical exposure of <1 second [30]. The particle concentration exposure (viral load) in this experiment (ρ_i_), is calculated using the volume flow rate of the nebulizer, the MMAD of the nebulized particles, the time of exposure and the volume of the enclosure.


ρi=NaCl(i)particleconcentration=5.64×105[cm−3]ϑ˙=volumeflowrateofnebulizedNaClsolution=0.25[cm3min]t=timeofexposure=20[min]ri=MedianmassaerodynamicradiusofNaClparticles=2.65[μm]V=VolumeofVFEexperimentalenclosure=1.135×105[cm3]ρi=∫0tϑ˙dt43πri3×1V (5)

The calculated particle concentration at >5x10^5^ cm^-3^ can be translated into the total exposure, which is more than 1200 times a typical exposure [30]. However, the results may need to be adjusted for respiratory particle size, longevity, climatic conditions etc.

## 3. Results

### 3.1. Particulate Filtration Efficiency (PFE)

**Fig 5:**
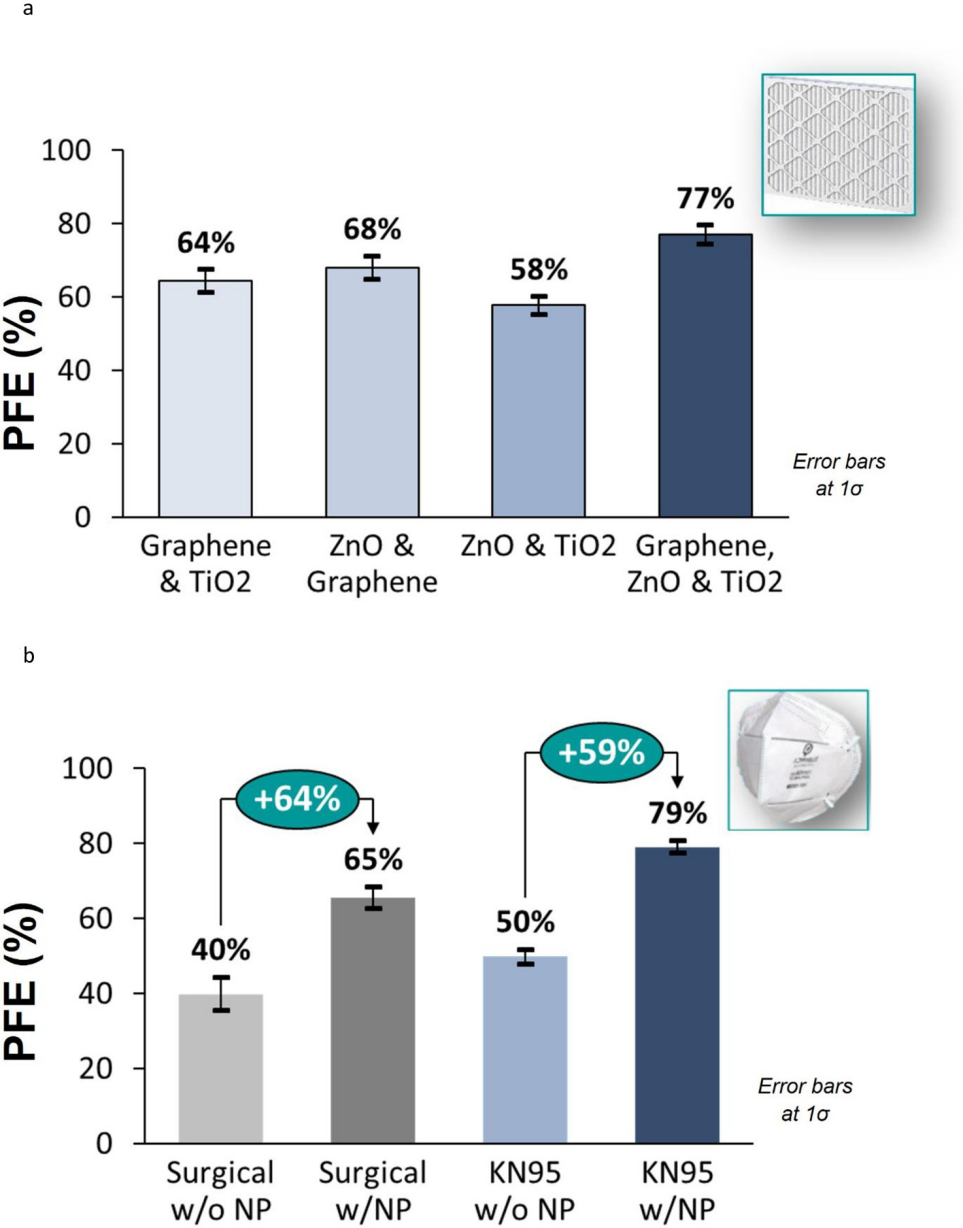
**a**. PFE on HVAC filters improved by optimization of different nanoparticle coatings. **b**. Nanoparticle coatings on surgical and KN95 masks improve their PFE by 60%.

PFE was optimized by varying the concentrations and the combination of different nanoparticles as applied on HVAC filters (Fig 5A). The PFE obtained was comparable to expensive filtration systems like ionic or HEPA [12,13,19]. When this optimized mixture was applied to regular surgical and KN95 face masks, there was a relative increase of approximately 60% in the PFE (Fig 5B) compared to uncoated masks. The absolute PFE for the nanoparticle coated KN95 masks, approximately 80%, are also similar to that of the coated HVAC filters. KN95 masks were found to be better than surgical masks in PFE, as is commonly known.

**Fig 6:**
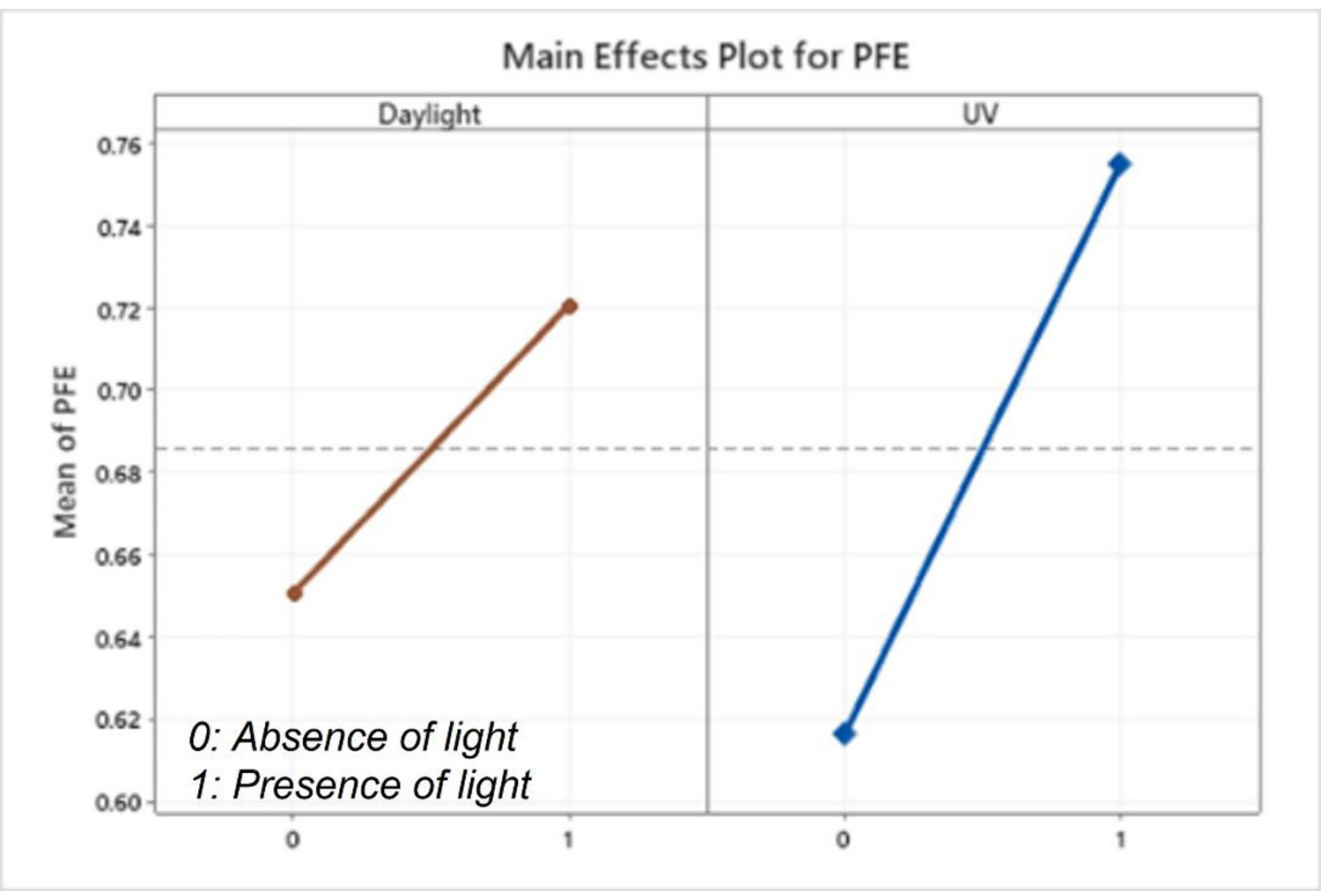
Photocatalytic activation of nanoparticles enhances PFE by ~7% in daylight and ~13% in UV light.

The main effects plot from the Analysis of Variance (ANOVA) analysis, as seen in Fig 6, shows the statistically significant dependence of PFE with light exposure, due to the photocatalytic activation of nanoparticles. PFE increased by 7% with exposure to daylight and by 13% in presence of UV light. Amongst the nanoparticle combination used in this study, TiO_2_ is known [31] to have photo-catalytic properties due to low bandgap (3.2 eV) between the conduction and valence bands, and CuO (bandgap of 1.7 eV) enhances photo-catalytic activity by further lowering the heterojunction bandgap (1.9 eV) in a CuO-TiO_2_ combination [31].

### 3.2. Nanoparticle Retention Efficacy (NRE)

**Fig 7:**
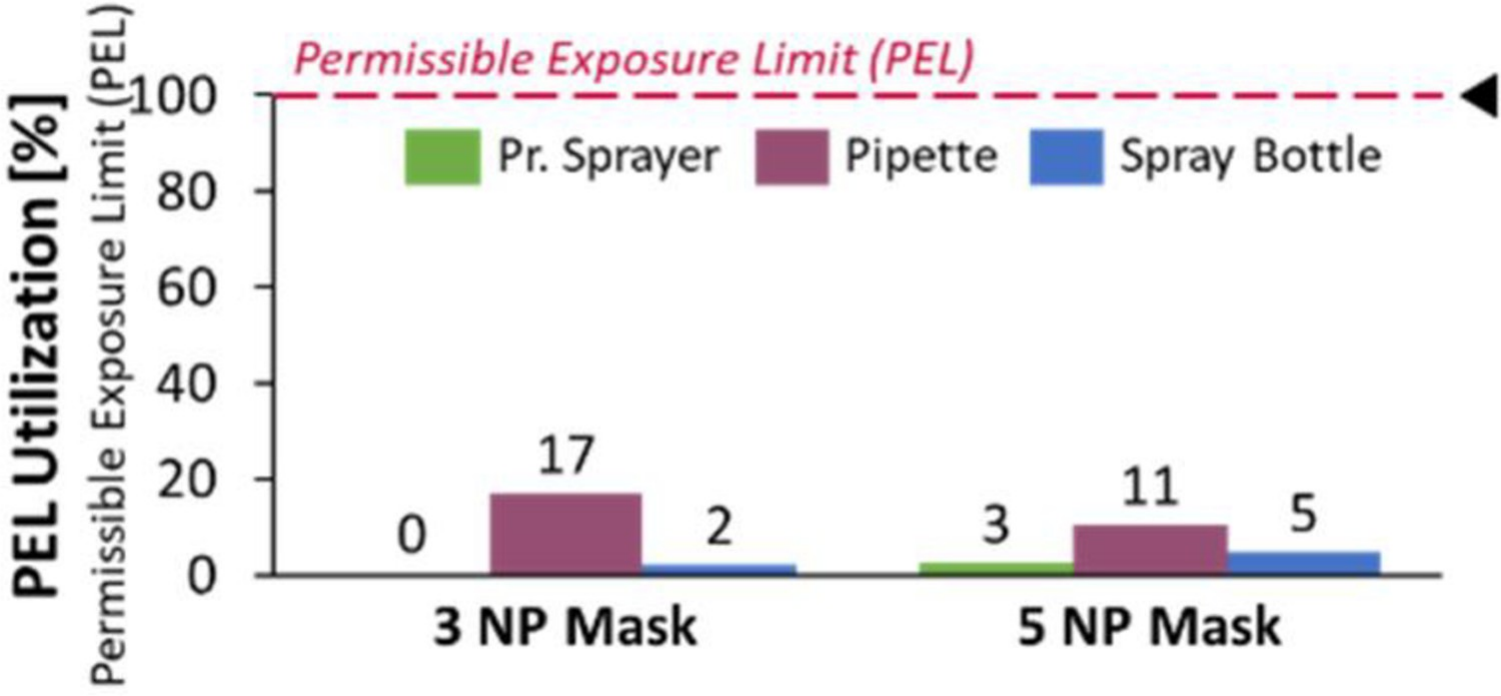
Pressurized sprayer yielded best deposition with lowest PEL utilization.

The NRE testing was performed to evaluate the different nanoparticle deposition methods against their PEL utilization. It was seen that the pressurized spray deposition method yields the best retention efficacy or the lowest PEL Utilization, as per (3) above, as compared to the pipette or spray bottle application methods, as can be seen in Fig 7. The pipette application method has the most PEL utilization, most likely due to its difficulty in controllability and inherent inconsistencies in this application method.

**Fig 8:**
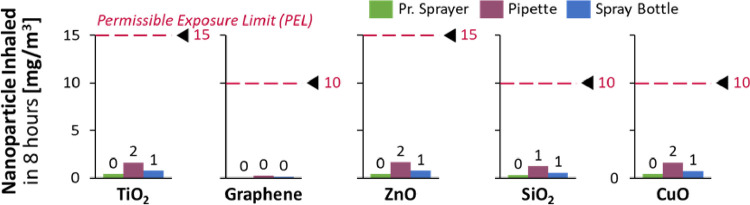
The dislodged nanoparticles are well within their Permissible Exposure Limits.

The embedded nanoparticles were evaluated against their individual Permissible Exposure Limits (10mg/m^3^ for Graphene, SiO_2_ and CuO and 15 mg/m^3^ for TiO_2_ and ZnO), as per OSHA standards [24]. All the nanoparticles were found to be well within their exposure limits (Fig 8) and the pressurized sprayer yielded the minimum dislodgment of nanoparticles, and the best Nanoparticle Retention Efficacy.

### 3.3. Virus Filtration Efficiency (VFE)

**Fig 9:**
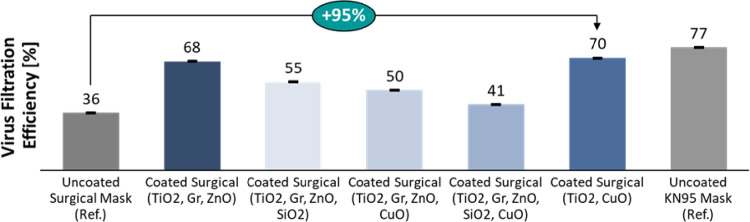
95% improvement in VFE with nanoparticles as compared to an uncoated mask. The small error bars (at 1σ) indicates measurement repeatability.

The VFE testing was done on regular surgical masks embedded with different combinations of nanoparticles and it was found that the nanoparticles were effective in almost doubling the virus filtration efficiency (Fig 9). The TiO_2_ and CuO combination demonstrated the best virus filtration efficiency due to their photo-catalytic properties, as described above. The downward trend of VFE with increasing nanoparticle content is explained due to the surrogate method of experimentation applied here. The biocidal and antiviral capabilities of the metal oxide nanoparticles like ZnO and CuO are not fully utilized by using NaCl as a surrogate. On the other hand, the probability of nanoparticle dislodgement increases with the increase in nanoparticles on the mask, while being bombarded by the larger NaCl particles. Hence, the masks with increasing nanoparticle concentrations tend to show a decreasing trend of VFE.

**Fig 10:**
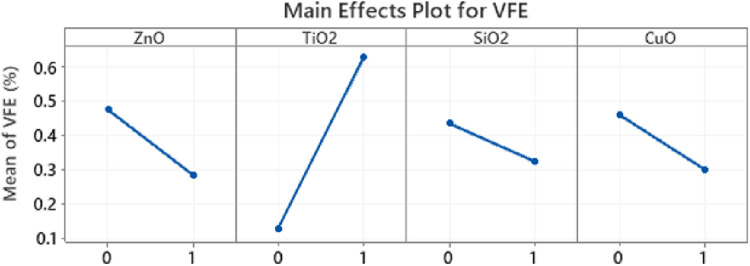
TiO_2_ main contributor to VFE improvement while the contribution of other nanoparticles inconclusive in this surrogate testing with NaCl.

The contribution of the individual nanoparticles to the improvement in VFE was tested using the ANOVA method (Fig 10) and it was demonstrated that TiO_2_ was most effective in improving VFE (p-value<0.05). The impact of other nanoparticles was inconclusive (p-value>0.05) since their anti-microbial and virucidal properties could not be evaluated with the surrogate NaCl particles.

### 3.4. Pressure drop and breathability

The pressure drop measurement for masks is a critical functional parameter affecting the breathability and comfort in wearing the mask. The pressure drop can be calculated by using the Bernoulli Eq (6):

$$P_{1}+\frac{1}{2}\rho \upsilon_{1}^{2}+\rho gh_{1}=P_{2}+\frac{1}{2}\rho \upsilon_{2}^{2}+\rho gh_{2}$$ (6)

where *P* is the pressure, *ρ* is the density of the fluid, *υ* is the velocity, *g* is the gravitational constant, *h* is the height, subscript_1_ denotes upstream conditions and subscript_2_ is for downstream conditions. Under steady, incompressible, and frictionless flow along a streamline assumption with the same horizontal height; (7) can be simplified to the pressure drop Eq (6):

$$\Delta P=\frac{1}{2}\rho (\upsilon_{1}^{2}-\upsilon_{2}^{2})$$ (7)

**Fig 11:**
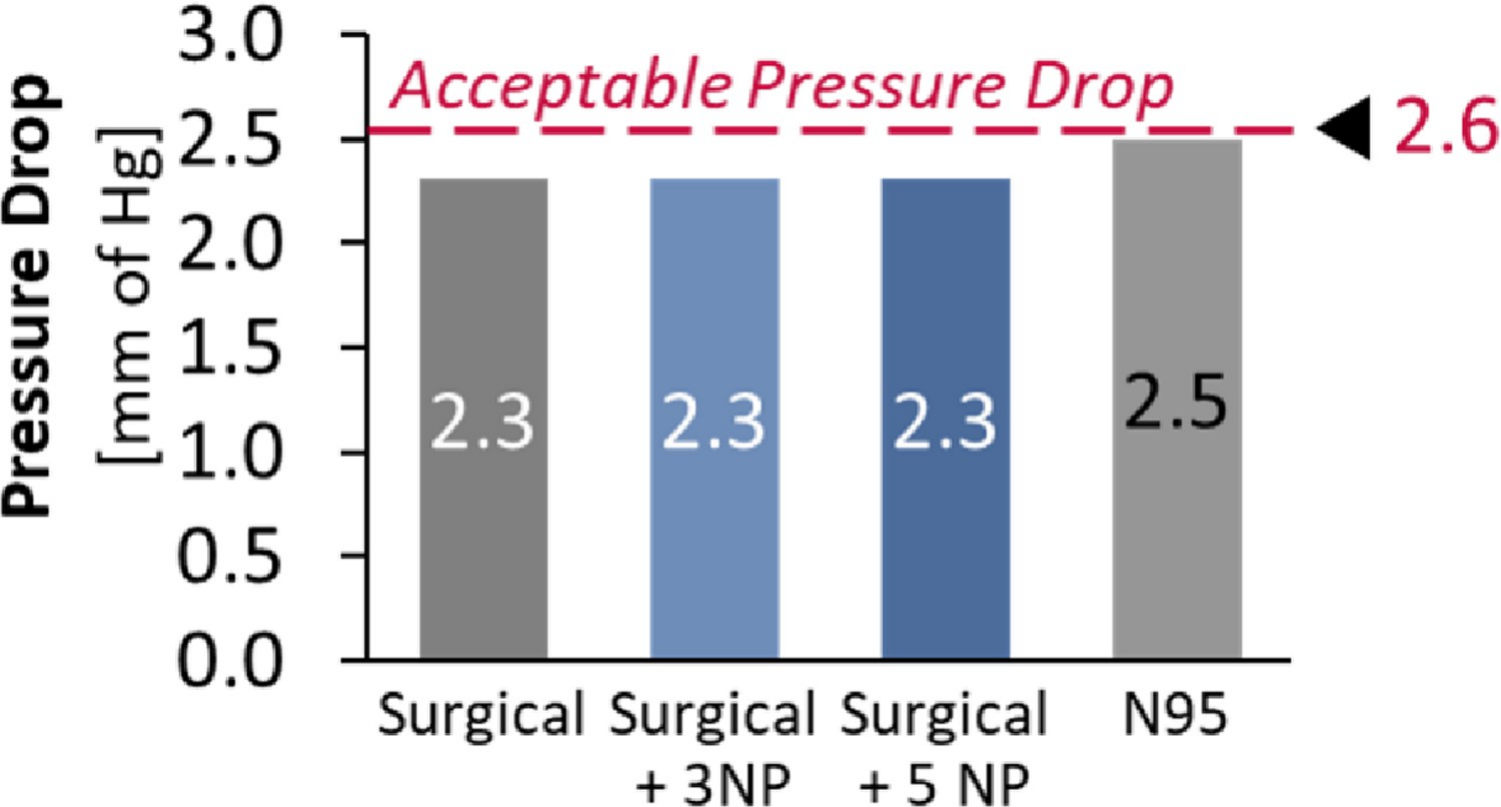
Pressure drop measurements on masks showed no significant change with addition of nanoparticles.

The pressure drop was measured using a manometer, with the tubes placed upstream and downstream of the masked mannequin. The results (Fig 11) indicate that all the measured masks were within the acceptable guidelines of the pressure drop requirements as specified by the CDC [32].

Furthermore, the deposition of nanoparticles did not have a significant or measurable impact on pressure drop of the masks. This minimal impact is expected, since the size of the nanoparticles (15–45 nm) are significantly smaller than the pore size of the masks (typically 10–90 μm [33]) and hence do not cause a significant blockage effect that could have an impact on pressure drop.

### 3.5. Measurement uncertainty

**Fig 12:**
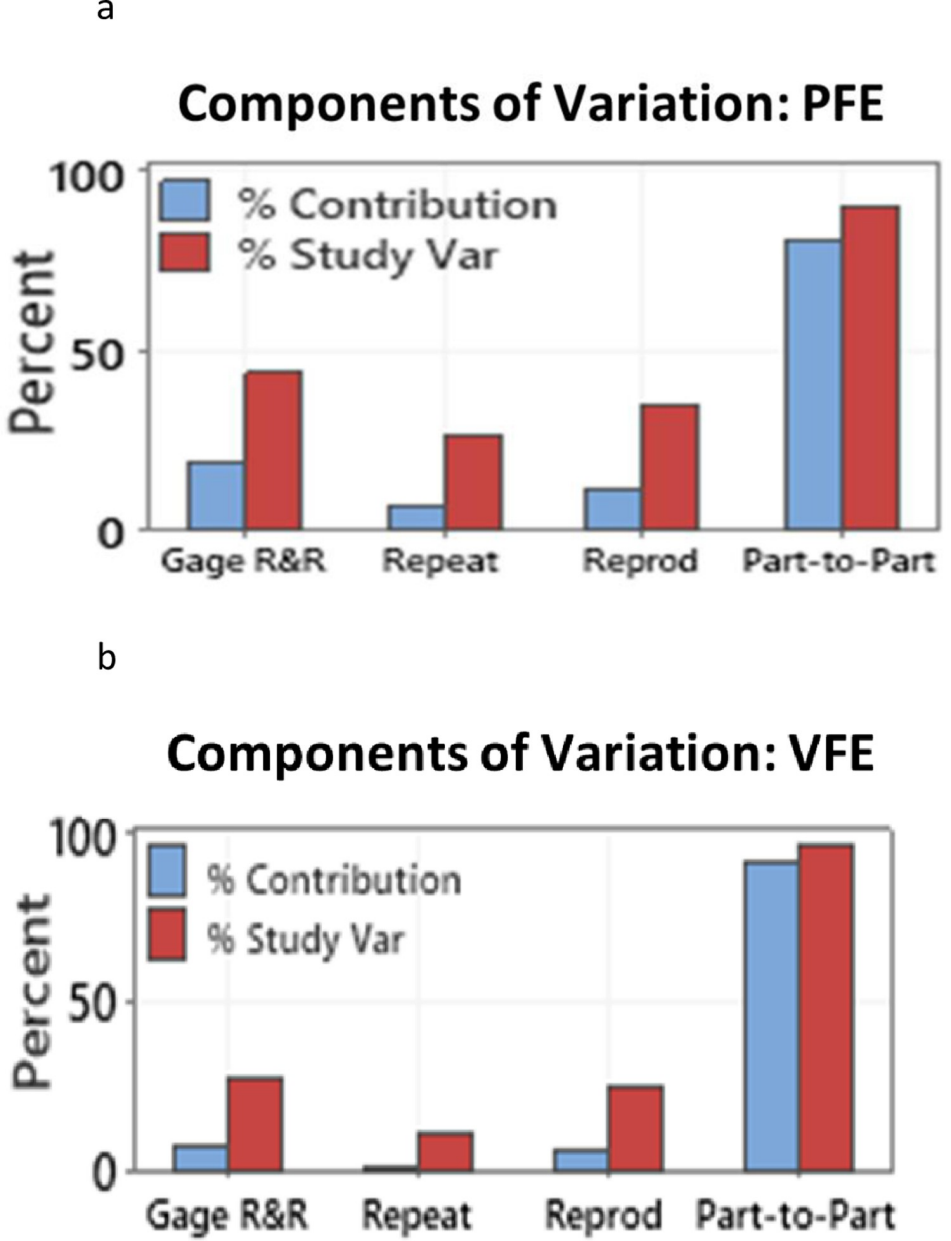
**a.** The Gage R&R analysis of the PFE experimental set-up indicated acceptable levels (7%) of measurement uncertainty. **b.** The Gage R&R analysis of the VFE experimental set-up indicated acceptable levels (8%) of measurement uncertainty.

A statistical repeatability and reproducibility study (Gage R&R) was used, using Minitab analytical software, to determine the measurement uncertainty of the experiments. For the PFE set-up, four different mask types were tested with ten repetitions each, and on two different days. As shown in Fig 12A, 93% of the contribution was from ‘part-to-part variation’ which is contributed by the natural process variation of the different masks and their coatings while 7% of the variation is contributed by the repeatability (one mask tested multiple times) and reproducibility (one mask tested over different days). The total Gage R&R being 7% is deemed to be an acceptable measurement uncertainty [34]. The measurement uncertainty of the VFE experimental set-up was evaluated by repeating the test 10 times (repeatability) each on 3 different coated masks on 2 different days (reproducibility). The total repeatability and reproducibility contribution were found to be 8% of the total variation and within the 10% acceptable limit (Fig 12B). The Gage R&R analyses of both the experimental set-ups indicated that they are repeatable and reproducible.

## 4. Discussion/Conclusions

### 4.1. Filtration and nanoparticle interactions

Filtration in masks of sub-micron scale particles are typically characterized by four mechanisms–inertial impaction, interception, diffusion, and electrostatic attraction [32]. Particles having too much inertia due to size or mass are not able to flow around a filter fiber. This mechanism is responsible for collecting larger particles of around 1μm. Interception happens when particles follow the primary flow streamline and pass close (within one particle width) to a filter fiber and are intercepted by the fiber. This mechanism is responsible for collecting particles typically around 0.6μm in diameter. Small particles, typically lower than 0.2μm in diameter, are constantly bombarded by air molecules due to Brownian motion, which causes them to deviate from the airstream and come into contact with a filter fiber and captured by this diffusion mechanism. Electrostatic attraction is a method by which oppositely charged particles are attracted to a charged fiber. This collection mechanism does not favor a certain particle size. Majority of filters characterized as N95, have a charged electret layer which enhances their electrostatic attraction mechanism [32]. The increase in PFE by 60%, as compared to uncoated masks demonstrated in this experiment confirms that the chosen combination of nanoparticles improved these filtration mechanisms. The photo-catalytic activation of nanoparticles has been also demonstrated with the enhancement of PFE by ~7% in daylight and ~13% in presence of UV light.

### 4.2. Virucidal effects of nanoparticles

The nanoparticle coated masks improved VFE by 95% as compared to an uncoated mask. The TiO_2_ and CuO combination demonstrated the best virus filtration efficiency due to their enhanced photo-catalytic properties. However, the biocidal and antiviral capabilities of the metal oxide nanoparticles like ZnO and CuO are not fully evaluated by using NaCl as a surrogate. Bacteriophage surrogates [35], like MS2 (nonenveloped, with single stranded RNA) or Phi 6 (enveloped, with double stranded RNA) displaying structural features similar to SARS-CoV-2 (enveloped, single stranded RNA), will be used in future studies to validate these properties.

**Fig 13:**
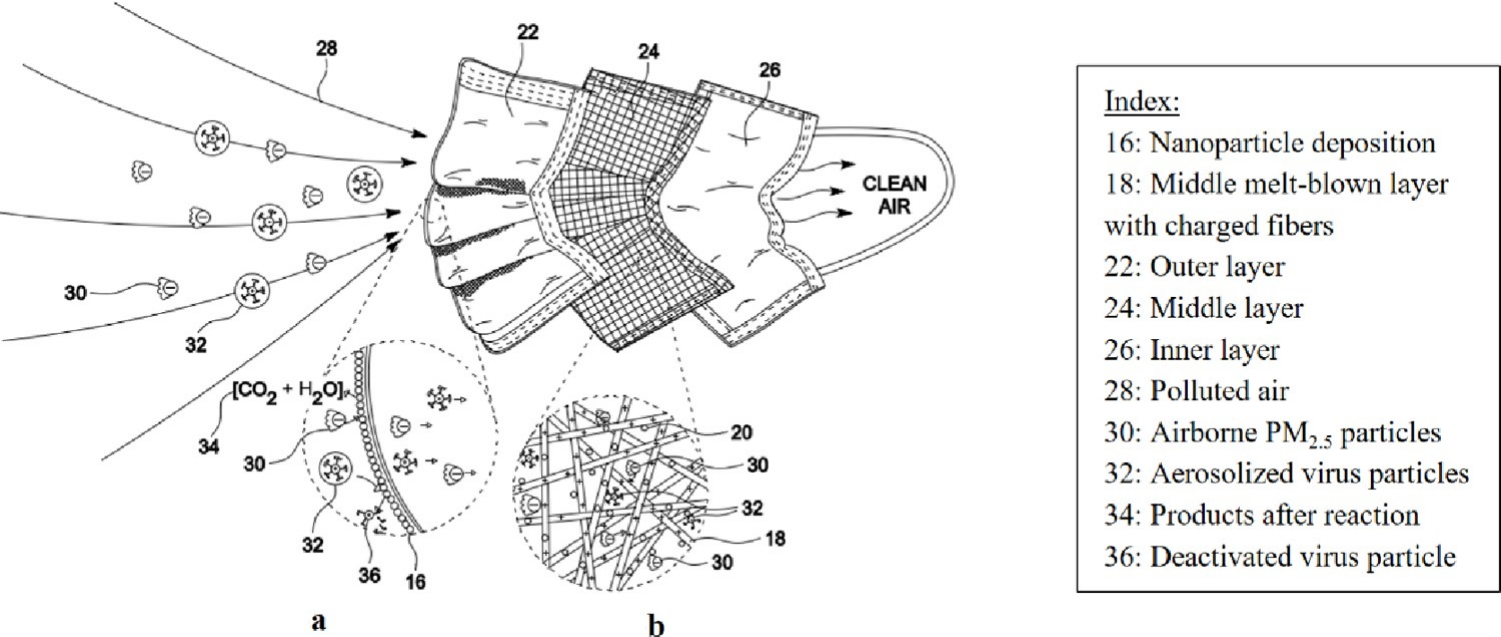
The nanoparticle coated mask is capturing and oxidizing particulate matter and virus particles with the aid of the nanoparticles embedded in its layers.

The schematic in Fig 13 shows a mask coated with nanoparticles on its outer layer, and some in its middle layer enabled by the pressurized deposition technique. The polluted air consists of airborne PM_2.5_ particles and aerosolized virus particles in mucus droplets. The embedded nanoparticles oxidize the PM_2.5_ particles into harmless carbon dioxide and water molecules (Fig 13A). The pollutants that can penetrate the nanoparticle coated outer layer are then adsorbed by the nanoparticles situated in the middle layer (Fig 13B), thus preventing them from entering the human body. The virus particles are similarly captured and oxidized by the metal-based nanoparticles by the generation of Reactive Oxygen Species which oxidize viral proteins and nucleic acids, such as the spike protein in SARS-CoV-2 virus, thus deactivating the virus particles.

### 4.3. Nanoparticle safety and future applications

The safety of nanoparticle usage is of utmost importance and continues to be a subject of research worldwide [36,37]. The nanoparticles chosen for this study are known for their clinical safety and non-toxicity and are extensively used in cosmetic and biomedical applications e.g., pill coatings, sunscreens [38]. The risk of nanoparticles being dislodged from the mask and inhaled were evaluated and found to be within 3% of the permissible exposure limit, as per OSHA standards [14] (Figs 7 and 8). The spray deposition method aerosolizes nanoparticles with compressed air and enables them to penetrate the superficial layers of the mask and become embedded in the inner layers, hence having a high retention efficacy (Fig 7).

Nanoparticle coated masks and filters have several applications such as in heavily polluted cities in China and India, in forest fire prone areas such as in Australia and California and for enhancing firefighter equipment. With their antimicrobial capability, they can be used in personal protective equipment, textiles, and packaging. The simple application method makes this technology versatile and usable in air-conditioning and car-cabin filters and in industrial pollution control systems.

### 4.4. Cost analysis

**Fig 14:**
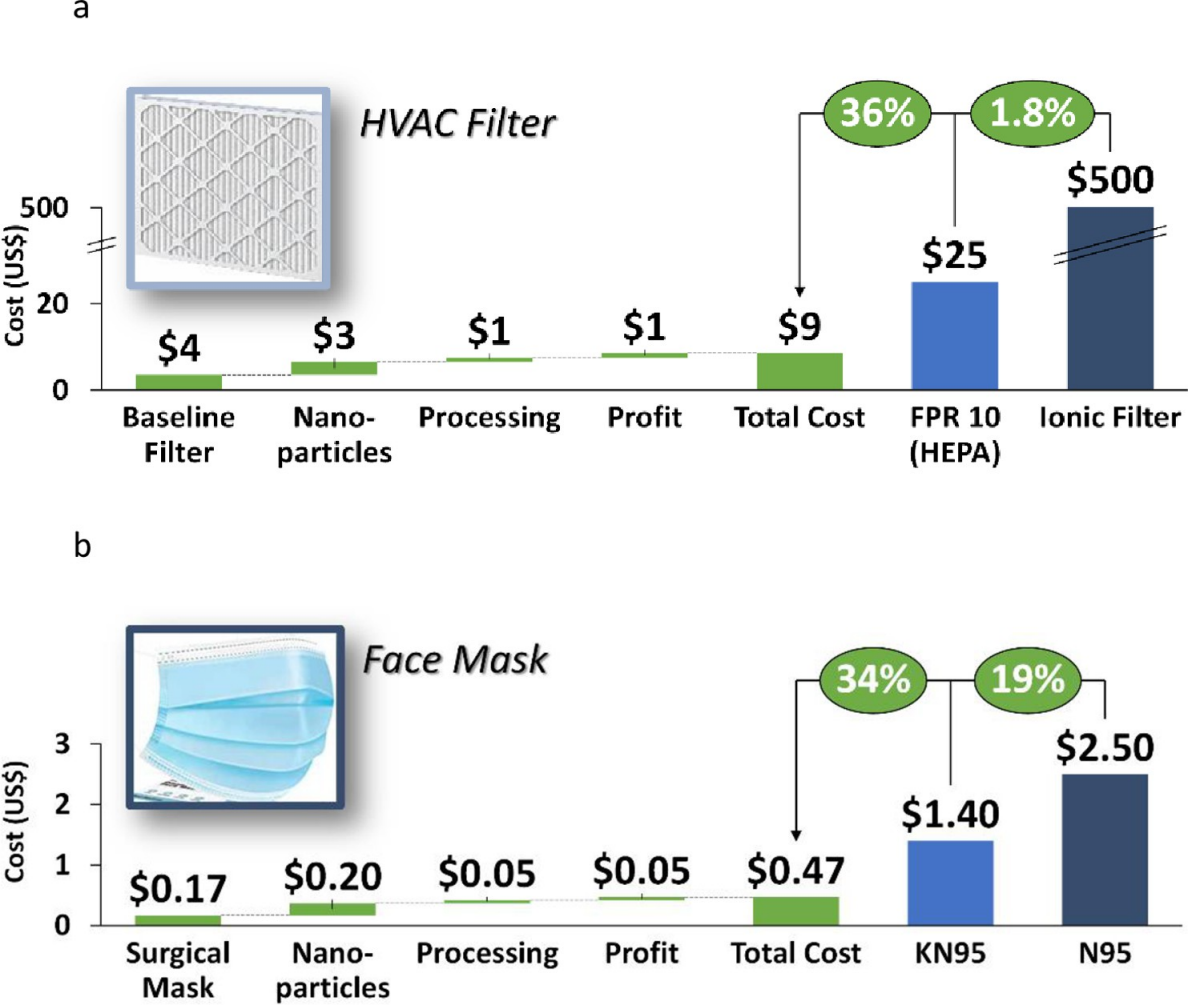
**a.** Nanoparticle coated HVAC filters are ~2% of the cost of an ionic filtration system and ~36% of the cost of a HEPA (FPR 10) HVAC filter. **b.** Nanoparticle coated surgical masks are ~19% of the cost of an N95 mask and ~34% of the cost of a KN95 mask.

A cost analysis was performed to evaluate the relative improvement in cost compared to high efficiency filtration devices. As seen in Fig 14A, the cost of the baseline filter is increased by the cost of the nanoparticles, processing costs, and profit. The resulting costs are still ~2% of the cost of an ionic filtration system and ~36% of the cost of a HEPA (FPR 10) HVAC filter, as reported in a previous study by this author [19]. Fig 14B illustrates a similar analysis on surgical masks. Even with the additional cost of nanoparticles, processing, profit, the total cost is ~19% of the cost of a N95 mask and ~34% the cost of a KN95 mask. Hence the nanoparticle coated filters and masks provide a cost-optimized alternative to expensive filtration systems while having similar efficiencies.

### 4.5. Conclusions

This experiment demonstrated that the chosen combination of nanoparticles provides an effective and safe solution for both particulate matter and viral particle filtration. The choice of the nanoparticles was based on their clinical safety and non-toxicity, and it was demonstrated that the dislodged particles were well within acceptable standards. The versatility and effectiveness of this filtration system makes it applicable in communities with limited resources and those with the highest risks of the deadly effects of air pollution and virus exposure. The significant correlation between air pollution and human fatalities due to respiratory illnesses caused by virus infections such as COVID-19 makes it essential for individuals to utilize abatement technologies such as nanoparticle coated filtration systems to save human lives.
